# Supplementary material for: Immunohistochemical expression of substance P in breast cancer and its association with prognostic parameters and Ki-67 index
Source: PLoS One. 2021 Jun 4;16(6):e0252616. doi: 10.1371/journal.pone.0252616 (PMC8177477; doi:10.1371/journal.pone.0252616)
Supplement: Attachment — Submitted filename: Response to reviewers.docx [file pone.0252616.s002.docx]

**May 17^th^, 2021**

**PONE-D-21-03600**

Dear Editor,

We want to thank you and the reviewers for the generous comments on the manuscript titled “**Immunohistochemical Expression of Substance P in Breast Cancer and its Association with Prognostic Parameters and Ki-67 Index**”. We have edited the manuscript to address all the comments.

Below please find our responses:

**A. Journal Requirements:**

When submitting your revision, we need you to address these additional requirements.

1. **Please ensure that your manuscript meets PLOS ONE's style requirements, including those for file naming. The PLOS ONE style templates can be found at**

https://journals.plos.org/plosone/s/file?id=wjVg/PLOSOne_formatting_sample_main_body.pdf and

https://journals.plos.org/plosone/s/file?id=ba62/PLOSOne_formatting_sample_title_authors_affiliations.pdf

**Response: Done**

2. Please provide additional details regarding participant consent. In the ethics statement in the Methods and online submission information, please ensure that you have specified (1) whether consent was informed and (2) what type you obtained (for instance, written or verbal, and if verbal, how it was documented and witnessed). If your study included minors, state whether you obtained consent from parents or guardians. If the need for consent was waived by the ethics committee, please include this information.

**If you are reporting a retrospective study of medical records or archived samples, please ensure that you have discussed whether all data were fully anonymized before you accessed them and/or whether the IRB or ethics committee waived the requirement for informed consent. If patients provided informed written consent to have data from their medical records used in research, please include this information.**

**Response: Done**

3. **We note that you have included the phrase “data not shown” in your manuscript. Unfortunately, this does not meet our data sharing requirements. PLOS does not permit references to inaccessible data. We require that authors provide all relevant data within the paper, Supporting Information files, or in an acceptable, public repository. Please add a citation to support this phrase or upload the data that corresponds with these findings to a stable repository (such as Figshare or Dryad) and provide and URLs, DOIs, or accession numbers that may be used to access these data. Or, if the data are not a core part of the research being presented in your study, we ask that you remove the phrase that refers to these data.**

**Response: The phrase that refers to the “data not shown” has been removed as it is not essential for this article.**

4. **We note that you have indicated that data from this study are available upon request. PLOS only allows data to be available upon request if there are legal or ethical restrictions on sharing data publicly. For information on unacceptable data access restrictions, please see** [**http://journals.plos.org/plosone/s/data-availability#loc-unacceptable-data-access-restrictions**](http://journals.plos.org/plosone/s/data-availability#loc-unacceptable-data-access-restrictions)**.**

**In your revised cover letter, please address the following prompts:**

4a) If there are ethical or legal restrictions on sharing a de-identified data set, please explain them in detail (e.g., data contain potentially identifying or sensitive patient information) and who has imposed them (e.g., an ethics committee). Please also provide contact information for a data access committee, ethics committee, or other institutional body to which data requests may be sent.

**4b) If there are no restrictions, please upload the minimal anonymized data set necessary to replicate your study findings as either Supporting Information files or to a stable, public repository and provide us with the relevant URLs, DOIs, or accession numbers. Please see http://www.bmj.com/content/340/bmj.c181.long for guidelines on how to de-identify and prepare clinical data for publication. For a list of acceptable repositories, please see** [**http://journals.plos.org/plosone/s/data-availability#loc-recommended-repositories**](http://journals.plos.org/plosone/s/data-availability#loc-recommended-repositories)**.**

**Response: The data has been uploaded as supplementary file (S1 File). This is notified in the statistical analysis subsection of materials and methods line 147 of the revised manuscript.**

We will update your Data Availability statement on your behalf to reflect the information you provide.

[Note: HTML markup is below. Please do not edit.]

**B. Reviewers' comments:**

Reviewer's Responses to Questions

**Comments to the Author**

**1. Is the manuscript technically sound, and do the data support the conclusions?**

The manuscript must describe a technically sound piece of scientific research with data that supports the conclusions. Experiments must have been conducted rigorously, with appropriate controls, replication, and sample sizes. The conclusions must be drawn appropriately based on the data presented.

Reviewer #1: Partly

Reviewer #2: Yes

**2. Has the statistical analysis been performed appropriately and rigorously?**

Reviewer #1: I Don't Know

Reviewer #2: Yes

**3. Have the authors made all data underlying the findings in their manuscript fully available?**

The [PLOS Data policy](http://www.plosone.org/static/policies.action#sharing) requires authors to make all data underlying the findings described in their manuscript fully available without restriction, with rare exception (please refer to the Data Availability Statement in the manuscript PDF file). The data should be provided as part of the manuscript or its supporting information, or deposited to a public repository. For example, in addition to summary statistics, the data points behind means, medians and variance measures should be available. If there are restrictions on publicly sharing data—e.g. participant privacy or use of data from a third party—those must be specified.

Reviewer #1: Yes

Reviewer #2: Yes

**4. Is the manuscript presented in an intelligible fashion and written in standard English?**

PLOS ONE does not copyedit accepted manuscripts, so the language in submitted articles must be clear, correct, and unambiguous. Any typographical or grammatical errors should be corrected at revision, so please note any specific errors here.

Reviewer #1: Yes

Reviewer #2: Yes

**5. Review Comments to the Author**

Please use the space provided to explain your answers to the questions above. You may also include additional comments for the author, including concerns about dual publication, research ethics, or publication ethics. (Please upload your review as an attachment if it exceeds 20,000 characters)

**Reviewer #1:**

1- **Please include the approval number of your institutional review board and the reason why you did not obtain form of consent from patients.**

**Response: The IRB approval reference number has been added as requested under a new subsection in the materials and methods section (lines 86-90). Due to the retrospective nature of the study and that all the data were fully anonymized, a consent form was waived by the IRB.**

2- In line 49 "targeted" would be better instead of "biological".

**Response: “biological” has been replaced with “targeted” as recommended.**

3- **In line 80 what do you mean by "depth of infiltration"?**

**Response: How deep tumor cells invade into the tissue, which is considered one of the prognostic factors in breast cancer.**

4- **How did you construct your tissue microarrays? What was the size of each core, how many cores did you take from each case? Which instrument did you use?**

**Response: More details were added to the TMA subsection (Lines 94-105)**

5- **For ER and PR positivity what is your threshold? How did you evaluate HER2 by immunohistochemistry or FISH or both of them? Though you mentioned that you obtained these results pathology reports, please give some more detailed information.**

**Response: The cut-off value for ER and PR positivity was set at 5%.**

**Her2/neu status was evaluated according to the guidelines of IHC. FISH for Her2/neu status was not performed at the pathology department at KAUH. The guidelines consider 0 or +1 as negative, +3 as positive. All cases of +2 were sent to a more specialized center to do FISH and the results received were added to the pathology report as either positive or negative.**

**The above information has been added to the materials and methods section lines 80-84.**

6- In line 161 you wrote that you have shown SP expression levels in Table 1 but they were shown them in table 2

**Response: The information are in Table 1 as cited, but for unknown reason the remaining of the table was omitted from the manuscript file. Thus we added the remaining of the table to the revised file.**

7- **In line 165 what do you mean by "negative expression of Ki67"?**

**Response: Negative expression means no staining for Ki-67 (score 0)**

8- **In line 203 it would be better if you write "prognostic staging" instead of "full staging" with mentioning the date of last AJCC.**

**Response: The term “full staging” was replaced by “prognostic staging” as recommended.**

**The date of the last AJCC was added as recommended.**

9- **You did not give detailed history of DCIS of your cases. Then you suggest that there is high expression of SP in women with history of DCIS. We do not know the time period between diagnosis of DCIS and invasive carcinoma, the site of DCIS, the type of treatment? It is quite speculative to draw such a conclusion.**

**Response: In almost all cases, DCIS is usually diagnosed at the same time in the same biopsy with invasive cancer. But in minority of cases, it is diagnosed alone. In such cases an immediate action to proceed with surgery is taken and duration does not exceed 2-3 weeks between both procedures**

10- **Please re-write last sentences of discussion of your manuscript based on your results. As you have not evaluated the relation between SP expression and prognosis and there is nothing about the diagnosis of breast cancer as well. The last sentences is not relevant to your findings.**

**Response: The sentences have been rewritten as recommended. (lines 309-312)**

**Reviewer #2**:

Substance P in breast cancer is a relatively under-researched area. The article is interesting with this aspect. However, some correction is required for it to be better.

1- **Line 80. "Clinicopathological data including age, gender, tumor volume, degree of histological 80 differentiation (well / moderate / poor, WHO), depth of infiltration, staging, and status of lymph nodes and distant metastasis, and hormone receptor (ER and PR) and Her2 / neu statuses were retrospectively collected from patients' medical charts. " I could not understand what "depth of infiltration" is used for.**

**Response**: **How deep tumor cells invade into the tissue, which is considered one of the prognostic factors in breast cancer.**

**2- Line 161. Table 1 should be changed to table 2.**

**Response: The information is in Table 1 as cited, but for unknown reason the remaining of the table was omitted from the manuscript file. Thus we added the remaining of the table to the revised file.**

**3- Figure 2 should be omitted.**

**Response: The figure has been omitted as recommended.**

4- **Table 2 design is done incorrectly. The percentage of substance P level for each molecular subtype should be given in the table. For example Her2 / neu enriched; 13/42 (30.95%), 29/42 (69.05%). The table should be rearranged as shown in the example. This error continues with stage, grade and other parameters. Additionally, the correction regarding these numbers (percentage etc.) should also be made in the discussion.**
**Response: Table 2 has been adjusted as recommended. The paragraph describing the numbers has been omitted as all the data are available in the table.**

**5- There is a difference in substance P expression level between which molecular subtypes are shown in Table 2. The p value for each molecular type should be given separately.**

**Response: Post hoc analysis by applying Bonferroni correction was performed. A figure to represent the results of the analysis are shown in an added figure (Fig. 3)**

**6- Is there any relationship between Ki67 and substance P? I could not see it in the text and in the summary. However, in the study with the title it is written that investigated this relationship. "Immunohistochemical Expression of Substance P in Breast Cancer and its Association with Prognostic Parameters and Ki-67 Index."**

**Response: No significant relationship was found between SP and Ki-67 expression levels. This was mentioned in the results section (revised manuscript; line 203), and discussion section (revised manuscript; lines 264-265), and we added a sentence to the conclusion section (line 309).**

**7- In the conclusion section, it should be written clearly in which molecular subtypes and at which stage the SP and ki67 levels are higher. The same is necessary for its relationship with ER, PR and lymph node metastasis. With this point of view, the summary should also be rearranged.**

**Response: Done.**

6. PLOS authors have the option to publish the peer review history of their article ([what does this mean?](https://journals.plos.org/plosone/s/editorial-and-peer-review-process#loc-peer-review-history)). If published, this will include your full peer review and any attached files.


If you choose “no”, your identity will remain anonymous but your review may still be made public.


**Do you want your identity to be public for this peer review?** For information about this choice, including consent withdrawal, please see our [Privacy Policy](https://www.plos.org/privacy-policy).

Reviewer #1: No

Reviewer #2: No

[NOTE: If reviewer comments were submitted as an attachment file, they will be attached to this email and accessible via the submission site. Please log into your account, locate the manuscript record, and check for the action link "View Attachments". If this link does not appear, there are no attachment files.]

While revising your submission, please upload your figure files to the Preflight Analysis and Conversion Engine (PACE) digital diagnostic tool, <https://pacev2.apexcovantage.com/>. PACE helps ensure that figures meet PLOS requirements. To use PACE, you must first register as a user. Registration is free. Then, login and navigate to the UPLOAD tab, where you will find detailed instructions on how to use the tool. If you encounter any issues or have any questions when using PACE, please email PLOS at [figures@plos.org](mailto:figures@plos.org). Please note that Supporting Information files do not need this step.
